# Cross-sector decision landscape in response to COVID-19: A qualitative network mapping analysis of North Carolina decision-makers

**DOI:** 10.3389/fpubh.2022.906602

**Published:** 2022-08-16

**Authors:** Caitlin B. Biddell, Karl T. Johnson, Mehul D. Patel, Raymond L. Smith, Hillary K. Hecht, Julie L. Swann, Maria E. Mayorga, Kristen Hassmiller Lich

**Affiliations:** ^1^Department of Health Policy and Management, Gillings School of Global Public Health, University of North Carolina at Chapel Hill, Chapel Hill, NC, United States; ^2^Lineberger Comprehensive Cancer Center, University of North Carolina at Chapel Hill, Chapel Hill, NC, United States; ^3^Department of Emergency Medicine, School of Medicine, University of North Carolina at Chapel Hill, Chapel Hill, NC, United States; ^4^Department of Engineering, East Carolina University, Greenville, NC, United States; ^5^Department of Industrial and Systems Engineering, North Carolina State University, Raleigh, NC, United States

**Keywords:** COVID-19, community health, cross-sector collaboration, decision-making, crisis response

## Abstract

**Introduction:**

The COVID-19 pandemic response has demonstrated the interconnectedness of individuals, organizations, and other entities jointly contributing to the production of community health. This response has involved stakeholders from numerous sectors who have been faced with new decisions, objectives, and constraints. We examined the cross-sector organizational decision landscape that formed in response to the COVID-19 pandemic in North Carolina.

**Methods:**

We conducted virtual semi-structured interviews with 44 organizational decision-makers representing nine sectors in North Carolina between October 2020 and January 2021 to understand the decision-making landscape within the first year of the COVID-19 pandemic. In line with a complexity/systems thinking lens, we defined the decision landscape as including decision-maker roles, key decisions, and interrelationships involved in producing community health. We used network mapping and conventional content analysis to analyze transcribed interviews, identifying relationships between stakeholders and synthesizing key themes.

**Results:**

Decision-maker roles were characterized by underlying tensions between balancing organizational mission with employee/community health and navigating organizational vs. individual responsibility for reducing transmission. Decision-makers' roles informed their perspectives and goals, which influenced decision outcomes. Key decisions fell into several broad categories, including how to translate public health guidance into practice; when to institute, and subsequently loosen, public health restrictions; and how to address downstream social and economic impacts of public health restrictions. Lastly, given limited and changing information, as well as limited resources and expertise, the COVID-19 response required cross-sector collaboration, which was commonly coordinated by local health departments who had the most connections of all organization types in the resulting network map.

**Conclusions:**

By documenting the local, cross-sector decision landscape that formed in response to COVID-19, we illuminate the impacts different organizations may have on information/misinformation, prevention behaviors, and, ultimately, health. Public health researchers and practitioners must understand, and work within, this complex decision landscape when responding to COVID-19 and future community health challenges.

## Introduction

Declared a pandemic by the World Health Organization on March 11, 2020 (1, 2), the coronavirus disease 2019 (COVID-19) continues to rapidly spread, resulting in over 6 million deaths worldwide as of March 2022 (3). COVID-19 has posed the most challenging and complex global health crisis in at least 100 years. Specifically, the complexity of COVID-19 has been characterized by: uncertain and rapidly changing information; interdependencies and feedback loops between decisions made by many individuals/organizations with different perspectives and their outcomes across organizational and sector boundaries; and time lags between policy changes and their ripple effects (4, 5).

In the United States, though federal guidance has been issued, the COVID-19 pandemic response has largely been implemented at the state and local level, involving ongoing decision-making by stakeholders across numerous sectors at these levels. Even before COVID-19, with the increasing recognition of the social and economic influences on health and health inequities, promoting local community health has demanded the involvement of numerous sectors operating at multiple levels of influence (e.g., individuals, organizations, policy-making).^6^ Health outcomes are thus collectively produced by a broad spectrum of stakeholders—defined as individuals and organizations with an interest in a given problem and its resolution (7, 8)—acting in accordance with their own goals, incentives, knowledge, and mental models of the problem at hand (9).

As a result, local community health promotion can be conceptualized as a complex system in and of itself, with interactions between different sectors resulting in feedback loops producing emergent properties across the entire system (6). In complex systems theory, emergent properties develop when systems evolve over time and develop effects that are different, or greater than, the sum of their parts (10, 11). In the context of community health, such properties could be understood as different dimensions of the community's health and safety (e.g., access to healthcare, safe environmental conditions, a positive culture of healthy behavior, etc.,). For this reason, studying the independent parts of a system, including decision-making within different sectors, is not sufficient to understand the emergent system properties influencing system outcomes. It is the collective decision-making of all stakeholders within the community that produces the overall level of community health.

Given the complexity of the COVID-19 pandemic, layered on top of the already complex landscape of local community health promotion, studying the local pandemic response demands a complex systems approach that recognizes the distinct yet interconnected stakeholder roles shaping decisions within and across organizational boundaries (12). In the context of a local pandemic response, stakeholders range from individuals deciding whether to wear a mask to local public health officials developing and communicating guidance around mask usage (13, 14). Given the influence that organizational policies had on individual-decision making during the pandemic, we bounded our study of the local pandemic response by focusing on organizational decision-makers, defined as individuals whose job responsibilities included making decisions with a substantial impact on the organization as a whole or individuals the organization serves.

Specifically, we sought to study the local cross-sector decision landscape emerging in response to the early-stages (first year) of the COVID-19 pandemic in North Carolina, a large, diverse state in the US with several metropolitan centers. North Carolina's local public health system is comprised of 85 local health departments, most commonly organized at the county-level (there are 100 counties in North Carolina). Historically, public health agencies have collaborated with county emergency management divisions for emergency preparedness and response—especially in response to hurricanes in the eastern region of the state—with several counties merging the role of public health preparedness coordinator with emergency management (15, 16).

We define the local cross-sector decision landscape in terms of *who* is involved in making decisions that affect community health, the *relationship* between decision-makers' roles and the types of decisions made, and the methods of *influence* between different stakeholders within the same community. Viewed through a complex systems lens, we considered decision-makers' organizations as *nodes* and the connections between them, formed through the decision-making process, as *interrelationships*. We conducted a network mapping-based qualitative analysis of organizational decision-makers in North Carolina.

Improving health, particularly amidst crises such as COVID-19, requires coordinating complex decision landscapes. This analysis illustrates a replicable approach to mapping and characterizing a complex organizational decision landscape. Within the context of the various organizational perspectives, priorities, and incentives involved in community health, the results of this analysis serve to inform decision-making by public health practitioners and researchers when responding to this and future infectious disease outbreaks, as well as other complex public health challenges that require system-level coordination.

## Materials and methods

### Sample description and recruitment

Defining sectors as subdivisions of society that include similar types of agencies or organizations serving distinct functions (7, 17), we interviewed state and local decision-makers from nine sectors: **business** (*n* = 4; small business owners, real estate agent, technology company director; B1-B4), **non-profit organizations** (*n* = 3; senior director, vice presidents (VP) of operations and risk management; NP1-NP3), **county government** (*n* = 4; county managers, director of social services; G1-G4), **healthcare** (*n* = 5; directors/VPs of healthcare associations, systems engineer, director of student health; H1-H5), **local public health** (*n* = 5; local health directors; PH1-PH5), **public safety** (*n* = 7; emergency managers, county sheriffs; PS1-PS7), **religion** (*n* = 6; church pastors, member of church COVID taskforce; R1-R6), **education** (*n* = 7; principal, school board member, community college president, university vice president; E1-E7), **transportation** (*n* = 3; transportation planner and pedestrian coordinator, traffic safety engineer; T1-T3) (Table 1).

**Table 1:** Characteristics of organizations represented in interviews with local decision-makers (*N* = 44).

**Organization characteristics**	***N* (%)**
**Sector** ^*^	
Public safety	7 (16%)
Education	7 (16%)
Religious organization	6 (14%)
Local public health	5 (11%)
Healthcare	5 (11%)
County government	4 (9%)
Business	4 (9%)
Non-profit organization	3 (7%)
Transportation	3 (7%)
**Region of North Carolina**	
Eastern (Coastal Plains & Sandhills)	9 (20%)
Piedmont	23 (52%)
Western (Mountains & Foothills)	5 (11%)
Multiple regions	7 (16%)
**Rurality of county** *^†^*	
Metropolitan	32 (73%)
Non-metropolitan	4 (9%)
Multiple counties	8 (18%)

* Interviewees within each sector represented different types of organizations, Public Safety (County Emergency Services/Management, County Sherriff's Office); Education (Universities, Community college, Private & public grade schools, School board); Religious Organization (Church leadership); Local Public Health (Local Health Departments); Healthcare (Healthcare association/society, Private health system, University student health); County Government (County Management, County Social Services); Business (Real estate, Retail shop, Coffee shop, Technology company); Community Organization (Recreation & youth programming, Food distribution); Transportation (City Transportation, State Transportation).

† Based on 2013 Rural Urban Continuum Code (RUCC) classification scheme; RUCC <4, metropolitan.

Given the challenge of asking organizational leaders to meet during the early stages of the pandemic, we used a snowball sampling approach, starting with intentionally diverse decision-makers recommended by our research team and their cross-sector contacts. We then asked interviewees for referrals to decision-makers in related organizations who may provide a meaningful and diverse perspective from their own. We interviewed 44 of the 120 potential interviewees contacted (37% response); four interviewees were previously known to one or more coauthors. Of those contacted who did not complete an interview, most did not respond to our email request. As such, we are not able to know the exact reasons for non-response. However, we suspect that this was due to the substantial competing demands of organizational leaders during the first year of the pandemic. No candidates explicitly refused to participate due to hesitation surrounding the study objectives. We determined sample size by reaching thematic saturation across sectors and ensuring at least three interviews within each sector. While the interviewees do not represent an exhaustive list of organizations responding to the pandemic, the objective of our sampling approach was to recruit decision-makers from diverse organizations and ensure representation across sectors and the state of North Carolina.

### Interview procedures

Three members of the study team (KTJ, MDP, KHL) developed the semi-structured interview guide following a review of decision theory literature and iteratively revised it during the first three interviews (Supplemental Appendix 1). One member of the study team (KTJ), a graduate research assistant with qualitative interview experience and visible racial and gender privilege, conducted semi-structured interviews using a secure web-based video-conferencing platform. All 45–60-min interviews were recorded and transcribed by an external audio to text automatic transcription service. Transcripts were cleaned and de-identified by members of the study team prior to analysis. Interviews were conducted between October 2020 and January 2021, during which North Carolina experienced a surge in cases, with daily COVID-19 hospitalization counts increasing from ~900 in early October to almost 4,000 in January. North Carolina began administering vaccines in mid-December 2020, however widespread distribution did not begin until late-January (18).

We asked interviewees about their perceived individual and organizational roles in the COVID-19 pandemic response. Interviewees were then prompted to reflect on the key decisions that their organizations made in response to the COVID-19 pandemic in the first two months (February and March 2020) and at the time of the interviews (October 2020 through January 2021), including decisions they anticipated having to make in the near future. In discussing each key decision, we probed interviewees on the other stakeholders (within and across sectors) influencing or contributing to their decision-making process. Interviewees were also asked about the decision-making context (e.g., community beliefs), inputs (e.g., data and scientific guidelines), and processes (e.g., decision-making systems) used by their organizations. Responses to these questions were analyzed and reported separately (*manuscript under review*). This study was determined to be exempt from review by the UNC Institutional Review Board (#20-2087).

### Qualitative analysis

We employed conventional content analysis to derive themes from the qualitative data (19). Using an inductive, iterative coding approach, we outlined a general codebook structure stemming from the semi-structured interview guide (Supplemental Appendix 1). We allowed interview codes and themes to emerge as two independent researchers (CBB, KTJ) coded each transcript using MAXQDA software (see Supplemental Appendix 2 for final codebook) (20). We analyzed excerpts within each code relating to the decision landscape (decision-making process codes analyzed separately), identifying major and minor themes. Decision-maker roles were coded to describe the individual's role in the organization, broadly speaking, as well as their role in the organization's pandemic response. Decisions identified by stakeholders were coded as belonging to one or more emergent categories: continuing/suspending in-person services, safety protocols, population served, testing/tracing, vaccination, physical resource allocation, human resource allocation. Within each decision category we analyzed excerpts by sector, identifying key decisions and documenting the interrelationships between decision topics across sectors. To explicitly analyze the interrelationships across sectors resulting from collaborative decision-making processes, we coded for examples of collaboration between organizations, defined as either mutual (both organizations benefitting) or dependent (one organization relying on another for either resources or information). We defined collaboration broadly as two or more entities involved in a joint venture or decision-making process (21, 22). Further, we coded for instances in which interviewees described perceiving the behavior of other local organizations and institutions as indirectly influencing these decisions, another form of interrelationships between stakeholders. The Consolidated Criteria for Reporting Qualitative Research (COREQ) checklist was used to guide our reporting of the qualitative analysis and results (23).

### Network mapping and analysis

We used Kumu, an online platform for organizing complex data, to develop a network map of within- and cross-sector organizational collaboration that formed in response to the COVID-19 pandemic in North Carolina, as described by the decision-makers we interviewed (24). Network mapping is a complex systems method intended to describe and visualize the roles, power dynamics, and relationships between stakeholders in a bounded system (25, 26). Using data from the collaboration codes described above, we first developed a matrix (with sectors along each axis) detailing all instances of collaboration described in interviews. We then inputted this information into Kumu, with organization types as nodes (color-coded by sector) and collaboration illustrated through connections (between two or more nodes). After building the full network map, we used functionality within Kumu to calculate two network metrics: *degree* and *closeness*. *Degree* is a measure of the total number of unique connections attached to each node and is used for identifying frequently-connected local organizations, or hubs, in the network. *Closeness*, quantified on a scale of 0–1, is a measure of how close each node is to other nodes in the network, accounting for the entire network structure, rather than only direct connections (as is the case with *degree*) (24).

## Study results

Of the 44 stakeholders interviewed, the majority represented organizations serving constituents within a single county (primarily metropolitan), and constituencies ranged from several hundred to over 1 million (Table 1, Supplemental Table 3). As key informant interviewees provided organizational perspectives, individual characteristics could not be disclosed. Themes (presented below) emerged within each of three domains comprising the COVID-19 pandemic response decision landscape: (1) Perceived organizational roles, (2) Key decisions, and (3) Interrelationships between organizations (Table 2). These themes describe who was involved in making and informing decisions, in what context decisions were made, and the complexity of this decision landscape across sectors.

**Table 2:** Decision landscape themes and representative quotations.

**DOMAIN (Themes)**	**Representative quotations**
**Roles**	
Necessity of balancing established organizational mission with newly imposed responsibility for employee/community safety	“*Probably our primary role would be to find a way to continue to serve the population in a safe way. That*'*s I think our primary response is how can we continue to serve, but in a way that is safe and gives confidence to folks to be able to continue some of the necessities of like, I mean we did a lot of essential service work, we do a lot of work for essential service employees. And so, we have to figure out how to serve that niche in a way that is safe and responsible. And so I would say continuing our service in a way that continues to protect the people we serve.*” *(NP3, Non-profit Org.)*
Navigating organizational vs. individual responsibility for reducing COVID-19 transmission	“*So I was challenged with the task and the responsibility of putting out videos and contacting the community asking them,* “*No. Hey listen, this is very serious.*” *And as a community leader here hoping against hope that they took me seriously. I also had to address some erroneous thinking on their part especially the thinking of,* “*I*'*m going to put my faith in God and I*'*m going to let God take care of me.*” *… We don*'*t place our responsibility on God. This is a collaboration and God will help us, but he does not dissolve us of our own responsibilities for ourselves.*” *(R4, Religion)* “*Our role became in an education and empowerment bent. It*'*s a personal expectation, one, to protect yourself, and two, to comply with it. To have the right tools and understand the systems and systems can have number of connotations, but the systems that impact you on a macro level, our job was really to empower and inform as well as make available resources.*” *(PS7, Public Safety)*
**Key decisions**	
How to translate public health guidance into given organizational context	“*I closed the interior of the space for 5 months, set up at the front door a walk-up counter... And I kept it that way much longer than the governor required, just because I needed to be confident that I could keep everyone safe, and that people were on board with protecting one another and not just adhering to some rules that I established … but wanting to be on the same team with protecting one another. It took a while to get there.*” *(B4, Business)*
When to institute, or loosen, public health restrictions	“*…through contact tracing and through our case investigation, we started also identifying some hotspots where we started seeing patterns in transmission…based on that data, we mobilized our testing resources out there to be able to provide onsite testing to reach a broader, wider number of people and maybe people that wouldn*'*t have necessarily come to our facility to be tested…*” *(PH2, Public Health)*
How to holistically address downstream pandemic impacts	“*…early on, especially in March, the decision was a health risk-based decision. How many people can we save from being sick? … But I think now, the decisions that are being made are more about the social disruption. And by that, I mean, the economic disruption. This pandemic is costing us lives, yes. But it is costing us financial well-being, and mental health well-being and all those other well-beings, right? Especially in college age individuals. For college age individuals… they*'*re not getting the health impacts that the 60 and older age group is facing... They*'*re getting the life disrupters.*” *(H5, Healthcare)*
**Interrelationships**	
Necessity of collaboration between organizations and stakeholders across sectors	“*…we have this local company that*'*s been here for almost 100 years, that charter, they do charter buses for weddings and for high school football games and things like that…So they were really close to going out of business, they had laid off pretty much all of their staff. And so when the city contacted them and said,* “*Hey, would you be willing or interested in helping us drive transit?*” *… And so very quickly, they pivoted and trained with us in like a week and learned our transit system, and were picking up passengers and charter buses…it ended up being a very mutually beneficial situation. And I think the city saved them from going out of business and they really saved a lot of our riders too.*” *(T1, Transportation)* “*Our EOC [Emergency Operations Center] was activated and we pulled in all your typical emergency services but then we stood up a health and human services branch that specifically focused on food insecurity, sheltering, and business recovery. Those were three big pieces out of the emergency operations center that we developed inter-agency working groups. It wasn*'*t just city, it wasn*'*t just the county. It was using volunteer organizations, faith-based organizations, non-governmental organizations and using their expertise, using their manpower, personnel, and the resources they could bring to help this entire thing together.*” *(PS3, Public Safety)* “*It was more or less like our emergency management partners, who have been fantastic partners, recognizing how big this was going to become, and talking with their partners in emergency management throughout the state, and particularly throughout the region, and really seeing where other counties were stubbing their toes, and just saying,* “*Hey, you need to be concentrating on public health, and allow us to deal with the frame. We*'*ll continue to work together with the understanding that nothing that we can do, pretty much, can be done without you giving us the okay because this is a public health pandemic.*” *(PH1, Public Health)*
Centrality of local health departments in the local pandemic response	“*We have our health director, she*'*s basically responsible and she*'*s the information liaison if you will for COVID-19. We, me and the board, we weren*'*t out trying to vet the data or peer review it or any of those kind of things. But our health director was taking the data she received from the CDC, she was taking the information she received from the North Carolina Department of Health and Human Services, she was taking the models that they were using to create the guides that they were giving. We took them to be trusted sources.*” *(G3, Government)* “*The challenge for us right now is that everybody wants to reopen…so everybody wants us to review their plans…*.
	*Everybody*'*s trying to figure out a way to maneuver around the restrictions that are out there. And how to make the case for how they can do it better than anybody else can.*” *(PH5, Public Health)* “*…we*'*re stepping back further up-stream, we*'*re really trying to educate the community. Whether it*'*s standard media like newspapers and TV, with our social media outlets. We are working with our city with a $200,000 project, to work on offering education through our, especially into our African American and Hispanic community, to try to educate them about COVID and to prevent it.*” *(PH3, Public Health)* “*So, we engaged community leaders, which included municipal leaders, superintendents, community college president, our local university, the president and leadership staff, many other leaders. So, we engaged them. We also engaged first responders. We engaged the faith community, other folks who serve in congregant care settings…. we did that really early on*” *(PH2, Public Health)*
Influence of decisions made by surrounding organizations	“*Our science collaborative, our medical informatics specialists have said behavior deprives outcomes. And even as the metrics came through they said,* “*The metrics are the result of community action.*” *So where, and I think, you know [County] is fairly progressive in that way, and we*'*ve been pretty good on mask wearing, all that stuff. And they said to us when [County] opens, when [County] opens, when these others big school districts open, it*'*s going to change the numbers, so get ready for that.*” *(E5, Education)*

### Perceived organizational roles

Interviewees' perceived roles in the COVID-19 pandemic response informed the set of relevant decisions their organizations faced and how they balanced inherent competing priorities (e.g., constituent, staff, and community safety; physical, social, and emotional wellness) in the decision-making process (Table 3). Across all sectors, interviewees described the responsibility of continuing to run their organization's operations within the new legal and safety constraints of stay-at-home orders and mandated safety protocols. Non-profits, religious organizations, and county governments underscored the heightened need for their social services, viewing their role as responding to the social and economic consequences of the pandemic. Education and transportation similarly recognized the necessity of their services and viewed their role as ensuring these services were delivered in an altered form to ensure community safety. Healthcare associations saw their primary role as convening organizations for the purposes of knowledge sharing, personal protective equipment (PPE) allocation, and advocacy to the state. LHDs and emergency management had more central roles in the pandemic response, with communicable disease management and crisis response being core functions of these respective entities. County emergency management and LHDs worked together, with LHDs leading the local public health response and emergency management facilitating communication and logistics. Though the extent to which COVID-19-related roles departed from traditional organizational responsibilities varied by organization, the following themes emerged across sectors.

**Table 3:** Organization roles and key decisions among interviewees (*N* = 44).

**Sector (Organizations represented)**	**Perceived role(s)**	**Representative decisions**
**Business** (Real estate, Retail shop, Coffee shop, Technology company)	Continuing to meet original business mission while taking responsibility for keeping customers safe	Closed shop to public and built online business (Retail) Masking, distancing, and sanitizing requirements for customers and staff (All) Worked with governments and shipping companies on optimization (Tech)
**Non-profit organization** (Recreation & youth programming, Food distribution)	Managing operations and risk management; tension between increased need for services and the responsibility of keeping staff, volunteers and clients safe	Suspended ancillary services (e.g., education) to focus on food distribution (Food) Updated volunteer safety protocols in response to changing CDC guidelines (All) Convened non-profits to support virtual learning (Rec)
**County government** (County Management, County Social Services)	Ensuring the safety of staff and direct clients; anticipating community needs stemming from COVID-19 economic impacts	Implemented safety protocols for in-person county staff (All) Created new position to oversee food delivery for kids at home (SS) Leased new building to accommodate social distancing (Mgmt.)
**Healthcare** (Healthcare association/society, Private health system, University student health)	Healthcare associations: Convening organizations for knowledge sharing, PPE allocation, and advocacy to the state. Health system/Student health: Ensuring the safety of providers and patients, with an emphasis on PPE allocation and COVID testing	Championed stay-at-home policy in the community (Health System) Ensured continuity of care for students leaving campus (Student Health) Created PPE group purchasing system (Association)
**Public health** (Local health departments (LHDs)	Limiting disease spread (testing, tracing, vaccination); Guiding the translation of public health guidance into local organizational context; Educating the public; Convening and engaging community stakeholders	Issued stay-at-home order and mask mandate in advance of the state Reviewed safety protocols for local organization re-opening plans Orchestrated strike teams to address homelessness and food insecurity
**Public safety** (County emergency Services/Management (EM), County Sherriff's Office)	County emergency management: Facilitating communication and logistics for the public health pandemic response. County sheriffs: Ensuring the safety of staff and people under the care of law enforcement; enforcing executive orders	Decreased number of arrests to reduce detention center volume (Sherriff) Issued warnings for businesses not following protocol (EM) Forecasted PPE needed to run emergency operation center (EM)
**Religious Org.** (Churches)	Meeting the social and safety needs of church members and the broader community; being a source of trusted leadership; continuing to instill hope in community	Suspended (and in some cases, later resumed) in-person religious services Identified gaps in community social services and worked with other groups to meet those needs Partnered with LHD to host testing event
**Education** (Universities, Community college, Private & public grade schools, School board)	Promoting the well-being of students through continuing education; meeting social needs of students' families and surrounding communities; ensuring student safety	Transitioned to remote learning (All) Hired COVID coordinators at each school responsible for temperature and symptom checks (Primary, Secondary) Delivered laptops and hotspots to students (Primary, Secondary)
**Transportation** (City Transportation, State DOT)	Ensuring safety of citizens while using public transit, public spaces, and roadways	Transitioned public input sessions to be virtual (All) Hired private transportation company to supplement/avoid cutting routes (City) Lent businesses public space for outdoor dining (City)

#### Necessity of balancing established organizational mission with newly imposed responsibility for employee/community safety

Interviewees from all sectors prioritized customer, constituent, and community safety, often as a new responsibility in addition to their originally stated missions. For example, an interviewee from a non-profit dedicated to youth and recreational programming emphasized the challenge of carrying out this mission when they could no longer bring the community together in-person. In this case, the organizational mission and the responsibility for community safety were viewed as being in tension with one another; however, other interviewees viewed keeping their constituents safe as consistent with their original organizational mission, which became “*more urgent than ever before*” (R1, Religion). This responsibility also extended to the health of the broader community. “*The safer we are here, the safer folks are in the community*” (R2, Religion).

#### Navigating organizational vs. individual responsibility for reducing COVID-19 transmission

Given that many COVID-19 safety protocols required individual behavior change, interviewees acknowledged the limitations of their organizational roles in enforcing these measures. However, they underscored their role as being to educate and empower the public to uphold their personal responsibilities in mitigating COVID-19 spread. “*It's a personal expectation, one, to protect yourself, and two, to comply with it...Our job was really to empower and inform as well as make available resources*” (PS7, Public Safety). One pastor disseminated educational videos to combat misinformation—“*This is a collaboration and God will help us, but he does not dissolve us of our own responsibilities for ourselves*” (R4, Religion). The form of education varied, from ensuring that public health guidance was widely available to tailoring guidance to communities. Interviewees emphasized the importance of ensuring that constituents understood why public health measures were needed. Empowerment included leadership modeling public health behaviors and securing the resources, such as masks, to support community health-minded decisions.

### Key decisions

Fulfilling the roles described above involved decisions related to continuing or suspending in-person services, instituting safety protocols, allocating resources (human and physical), testing/screening, contact tracing, and vaccination. Interviewees described a decision ecosystem in which the consequences of one decision (whether related to viral transmission, economic impacts, or organizational realities) prompted the need for subsequent decisions. Further, given how quickly scientific knowledge and public health guidance were changing, interviewees were constantly faced with new decisions across domains. A full matrix of COVID-19-related decisions described is included in Supplemental Appendix 4 and summarized in Table 3. In analyzing the key decisions described by interviewees across sectors, the following thematic decision categories emerged.

#### How to translate public health guidance into organizational context

All interviewees made decisions to discontinue, or transition remotely, all non-essential in-person services in March 2020, informed by state and local stay-at-home orders. Though this was framed less as a decision, and more as a necessarily cautious response to the uncertainties of the pandemic, it prompted a cascade of decisions related to translating guidance into organizational contexts to maintain services/mission while ensuring employee and community safety. Decisions included distinguishing essential vs. non-essential personnel to inform remote work scheduling, securing PPE for essential personnel, and securing the technology necessary to support remote work. Even LHDs had to make internal staffing and protocol decisions, all while being propelled into a more central role than ever before. “*A big part of my workforce have children… How do we work and show up to serve the community while balancing the needs of what you're having to do at home?*” (PH4, Public Health).

In contrast, re-opening decisions were more contentious. While many strove to re-open, some decision-makers remained closed or instituted safety protocols beyond legal mandates. “*I needed to be confident that I could keep everyone safe, and that people were on board with protecting one another*” (B4, Business). However, pressure from community members to re-open grew over time. “*I've watched some of my colleagues at more conservative schools have to make decisions that they weren't 100% comfortable with, in terms of how rooms were organized, in terms of mask use …because of the pressure of their community*.” (E3, Education).

#### When to institute, or loosen, public health restrictions

While not all sectors were directly involved in testing, tracing, and vaccination, related decisions made by LHDs and emergency management influenced community transmission, and thus decisions about re-opening and safety protocols by organizations in other sectors. LHDs and EMs instituted contact tracing early on. “*To date, we believe that we maintained a seven-day rolling average of less than a hundred cases a day because we continue to do contact tracing*.” (PS4, Public Safety). LHDs and emergency management also implemented testing, often in partnership with external clinical entities; however, interviewees described challenges in carrying out these services equitably at scale. “*Contact tracing in most public health agencies wasn't fit for purpose, for the scale*” (B2, Business).

#### How to holistically address downstream pandemic impacts

A final category of decisions related to developing new or extending existing services to address social impacts of COVID-19 restrictions, such as homelessness and food insecurity. In some cases, this meant balancing infection risk with health risks of downstream consequences. Interviewees noted a primary tension in that efforts to “*dampen down COVID in our community are also the things that are putting some of our most vulnerable population at risk*” (PH5, Public Health). For organizations working to meet social needs, the recognition of heightened need motivated organization leaders to ensure services continued, even if processes had to change to keep staff, volunteers, and constituents safe. “*There's a whole litany of things that have kept us busier and have really proven the urgency and the significance of community-based and faith-based organizations*.” (R1, Religion).

### Interrelationships

The complexity and novelty of COVID-19 demanded the pooling of resources and expertise in decision-making, exemplifying the interrelationships between individuals, organizations, and resources within and across sectors. Given that organizational decision-makers were thrust into new roles and thus faced new decisions and competing priorities in response to the COVID-19 pandemic, they described turning to existing and new collaborations to navigate these complexities. Collaborative decision-making, as well as the influence of decisions made by other organizations on decision-making, showcase the complex interrelationships between individuals, organizations, and resources within and across sectors.

#### Network mapping results

**Figure 1:**
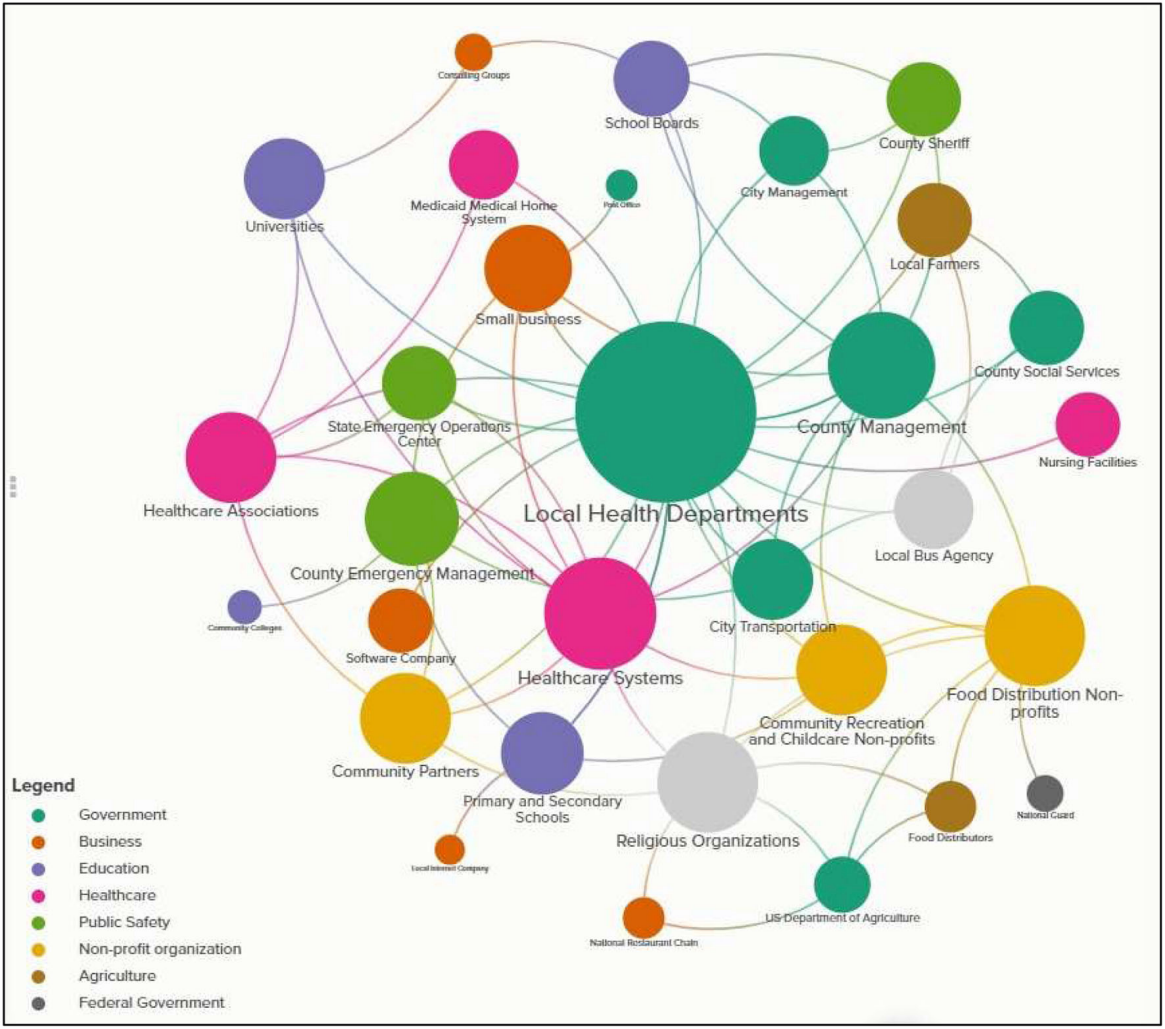
Network map of cross-sector partnerships formed in North Carolina's local COVID-19 pandemic response. This figure shows the network map developed from the collaboration described by interviewees. Each node in the map represents an organization type, color-coded by sector and sized by closeness metric (larger nodes more connected to other nodes in the network map). A full, interactive network map can be found (24).

Figure 1 shows the complexity inherent in the network map developed from the collaboration described by interviewees. Each node in the map represents an organization type, color-coded by sector and sized by closeness metric (larger nodes more connected to other nodes in the network map). Supplemental Appendix 5 presents complete network mapping metrics (i.e., closeness, degree). Additionally, a full, interactive network map can be found online. Hovering over individual connections and labeled loops will provide details about each collaboration represented in the map. Given that interviews were conducted among a small subset of all stakeholders involved in the local pandemic response decision landscape, and that interviews were limited to the most notable decisions interviewees were facing, this network map represents a subset of the connections and complexity involved in the complete decision landscape. Results from the network map, paired with qualitative analysis of excerpts coded as collaboration or the influence of other organizations, led to the synthesis of the following themes.

#### Necessity of collaboration between organizations and stakeholders across sectors

Interviewees described creatively responding to COVID-19-imposed challenges by forming new, and leveraging existing, collaborations among diverse stakeholder to prevent blind spots in decision-making. Three main categories of collaboration were identified: (1) **Public—Public**, particularly within sectors of local government (e.g., public health and emergency management co-leading the local pandemic response), (2) **Public—Private**, particularly government-initiated collaboration with non-governmental organizations (e.g., county social services partnering with community organizations to distribute COVID federal relief funds), and (3) **Private—Private**, particularly among businesses, non-profits, and religious organizations (e.g., local businesses partnering to deliver care packages to frontline workers). Interviewees universally described feeling that their collaborative capacity became stronger because of COVID-19, “*One of the positives that's going to come out of COVID is that we're going to have a more robust, cohesive, collaborative model of non–profits and organizations working together*” (R1, Religion). As measured in both *degree* and *closeness*, LHDs, healthcare systems, and county management were the most central actors in the decision landscape, documenting the high frequency with which they collaborated with other organizations in response to COVID-19 and their central role (Supplemental Appendix 5).

#### Centrality of local health departments in the local pandemic response

LHDs in our network map had a total of 24 unique connections (*degree*) and the highest *closeness* metric of 0.867 (Supplemental Appendix 5). As *closeness* is measured on a scale from 0 to 1, a *closeness* metric of 0.867 suggests that, of the organizations included in our analysis, LHDs had the most direct and indirect connections to other organizations in the network map. The next highest *closeness* metric was 0.633 (healthcare systems). On the whole, *closeness* metrics ranged from 0.356 to 0.867 with a mean of 0.51”.

**Figure 2:**
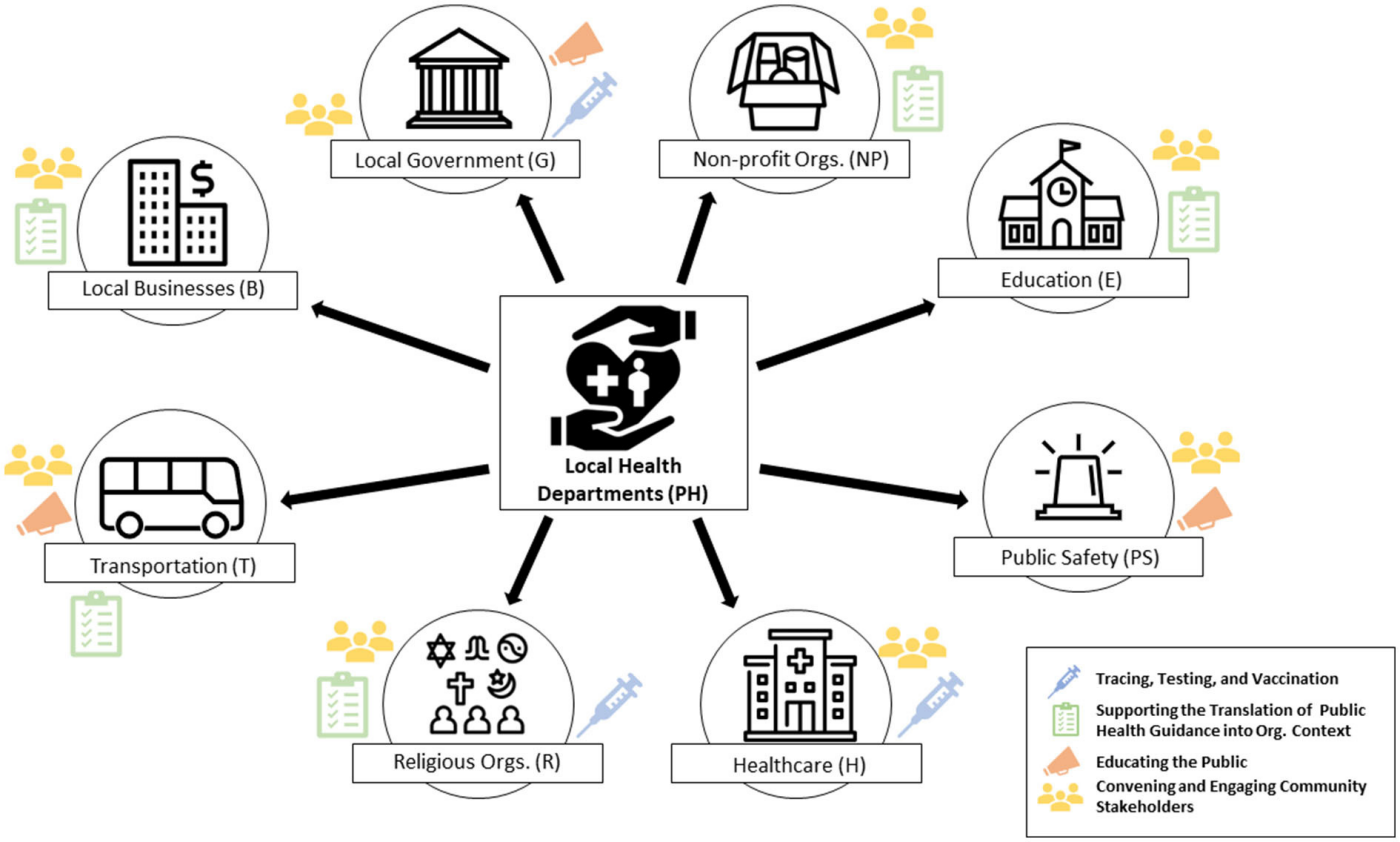
Central roles of local health departments in coordinating local COVID-19 pandemic response across sectors.

Central to many of the interrelationships described by interviewees, LHDs served a critical function in the pandemic response, both informing local decision-making and facilitating the implementation of higher-level decisions through collaboration with other sectors impacted by those decisions. LHDs served four primary collaborative roles: (1) Directly responding to the communicable disease outbreak (e.g., testing, tracing, vaccination); (2) Guiding the translation of public health guidance into local organizational contexts; (3) Educating the public; (4) Convening and engaging community stakeholders (Figure 2). Implementing a comprehensive pandemic response required collaborating with other sectors, such as hosting testing and vaccination events in parking lots. As described by an interviewee whose church volunteered as a test site, “…*the fourth Saturday of the month, for as long as they want, this will be the test site here. That's one of the ways that we're trying to help folks in the community*.” (R2, Religion). LHDs informed decisions at the crossroads between federal- and state-level guidance (e.g., mask mandates, distancing guidelines) and local organizations. They were viewed as “trusted sources” (G3, Government), providing tailored public health advice, visiting local businesses, and reviewing safety protocols. Educating the public required monitoring and reporting local COVID-19 trends through data dashboards and collaborating with leaders from other sectors to host press conferences and conduct educational campaigns. Lastly, LHDs were tasked with convening and connecting stakeholders across sectors to ensure the inclusion of diverse perspectives in addressing the economic and social determinants of health, creating “*better health through better partnerships”* (PH3, Public Health).

#### Influence of decisions made by surrounding organizations

Beyond the interrelationships resulting from explicit cross-sector collaboration, interviewees also described the impact of decisions made by the public and other surrounding organizations. As one interviewee noted in reference to the influence of community mask compliance and school district re-openings, “*metrics are the result of community action… If we change our behavior, it's going to change the numbers”* (E5, Education). In addition to influencing COVID-19 transmission trends, local decisions were described as influencing the feasibility of asking employees, volunteers, or customers to return in-person (e.g., Are schools open to provide childcare? Is public transportation running at full capacity?).

## Discussion

The COVID-19 pandemic has thrust decision-makers across sectors into new roles in a public health crisis response, creating a decision landscape with numerous actors and varying levels of coordination between them. Qualitative inquiry and network mapping and analysis allowed us to examine this cross-sector decision landscape through a complexity/systems thinking lens. The pandemic has forced the development of new decision-maker roles and competing priorities that decision-makers have navigated with limited, uncertain, and changing information. In response to the complexity of COVID-19, decision-makers engaged in both collaborative and semi-autonomous decision-making processes and depended upon new authorities, especially LHDs. In this resulting “polycentric” decision-making system, public and private actors worked across different centers of decision-making and at different scales to collectively produce their community's health during the pandemic (27). This study serves to (1) inform public health researchers, practitioners, and organizational decision-makers in how to navigate this and future complex, cross-sector population health challenges, and (2) illustrate a replicable approach to mapping and characterizing complex decision landscapes.

This study builds off prior work highlighting cross-sector responses to crises such as Hurricane Katrina and H1N1 (28, 29). It also extends prior applications of network mapping to other complex health challenges, such as serious mental illness (26) and community health promotion networks (25). However, this study is the first, to our knowledge, to use network mapping to investigate a cross-sector decision landscape in response to COVID-19. Several prior studies have investigated decision-making in response to COVID-19 within single sectors. These studies support the decision categories that emerged from our analysis, including decisions related to allocating resources (30), translating guidance into real-world organizational context (31), and addressing downstream social impacts (32). Our finding that cross-sector collaborations were critical components of the COVID-19 pandemic response builds upon several prior studies illustrating specific collaborations emerging in response to COVID-19-related needs, ranging from childcare for healthcare workers to local COVID-19 surveillance through school districts (33–36).

In line with our findings, prior work has emphasized the importance of community engagement in comprehensive pandemic responses and the necessity of communicating well (e.g., using accessible yet accurate language) with diverse stakeholders amidst changing, uncertain information (37). Challenges with community-based approaches, however, include balancing the need to respond quickly with the time it takes to meaningfully garner stakeholder perspectives (38). The need to navigate complex tradeoffs and often conflicting priorities within a community further underscores the importance of a cross-system governance or organizing structure with input from many stakeholder groups (32). Given the need to act quickly, communities should agree on such structures in advance of public health crises. Our analysis highlighted the importance of LHDs serving as what “Public Health 3.0” defines as a “chief health strategist” in coordinating the local pandemic response (39), working with other organizations directly and indirectly to govern the local public health system (40).

The decision landscape emerging in response to COVID-19 has implications for efforts to promote population health, beyond the immediate context of COVID-19. Though a global pandemic uniquely affects all individuals and organizations, other population health challenges operate within complex systems, influenced by multi-level determinants, ranging from individual action to social policy (41). This can create inconsistent priorities and decisions within communities that block progress. The role of stakeholders across sectors in the pandemic response, and the interrelationships between these sectors, support the growing call for the importance of cross-sector collaboration in promoting population health (7, 42, 43). Our findings further align with the vision of “Public Health 3.0” to expand the reach and scope of public health to “address all factors that promote health and well-being, including those related to economic development, education, transportation, food, environment and housing” (39). Public health leaders advocating for this broadened definition of public health have underscored that carrying out this vision successfully requires sustainable cross-sector collaboration, community engagement, and the application of a systems perspective to problem solving (44).

The “10 Essential Public Health Services”, updated in 2019 to include a focus on health equity, also reflect this reality, which considers the public health system to include not only public health agencies and healthcare providers, but also public safety, human services, and education, among other sectors (45). The decisions described in our analysis broadly fall into the three core domains of the essential services of public health: assessment (e.g., contact tracing, testing), policy development (e.g., implementation of executive orders, mobilizing community partnerships, educating the public to support effective policy change), and assurance (e.g., workforce maintenance, ensuring equitable access to services) (46). However, the COVID-19 pandemic has showcased that the centrality of equity in the revised essential services may still be aspirational. Disparities in COVID-19 morbidity and mortality rates by race and socioeconomic status underscore the need for system-wide decision-making that better prioritizes equitable access to health services, ranging from healthy living conditions to clinical care (47, 48). Additionally, the pandemic has highlighted the importance of the essential service, to “build and maintain a strong organizational infrastructure for public health,” moving forward (46). Bringing together the many sectors involved in the United States' fragmented public health system effectively and sustainably, beyond the immediate aftermath of a crisis, requires local foundational infrastructure supporting timely and comprehensive data collection (49); flexible funding mechanisms that recognize the necessity of cross-sector work in public health (50); and sufficient staffing capacity, particularly in response to the burnout of the current public health workforce (51, 52).

These findings should be viewed in the context of several limitations. While we were intentional in ensuring diverse representation of interviewees across sectors, organization type, and geography (across North Carolina), the sample does not represent an exhaustive list of organization types involved in the COVID-19 response. In all complex systems work, how system boundaries are defined has the potential to influence findings (8). Though we defined the bounds of the system under study based on geography (North Carolina) and organizational decision-makers, this system is too large to have a formal roster of all stakeholders involved. Results may have been different had we focused on a single community (region, city, or county) within the state, which would have allowed us to gain a more complete understanding of all stakeholders and their interactions. The snowball sampling technique employed increases the potential that the opinions uncovered were more homogenous than they would be otherwise. However, we were explicit when asking for recommendations that we were interested in uncovering a more complete and broader perspective on the subject. Thematic saturation was based on generalizable themes that emerged across sectors. Future research should investigate specific instances of cross-sector collaboration in more bounded systems, interviewing a complete roster of stakeholders involved, to gain a more detailed understanding of the role of power dynamics and competing priorities in influencing system dynamics. We hope that this study, which sought a broad boundary, will inform and standardize future efforts to study complex decision landscapes across diverse communities to learn what is similar and distinct.

The timing of interviews with respect to official guidance, transmission rates, and vaccination rollout undoubtedly influenced participant responses. We incorporated timing into interviews and analysis. Additionally, participant responses may be subject to self-report bias, given limitations of recall and the potential for selective reporting. As interviews lasted no more than an hour, it is not feasible to expect interviewees to recount every decision involved in their organization's pandemic response. As such, we asked interviewees to prioritize the key, COVID-related decisions that they perceived to be most important to their organization. Lastly, decision-makers willing to participate in public health research may have differed from those who refused in the extent to which they valued and trusted scientific information. However, participants described a range of perspectives on how they incorporated scientific information into decision-making.

This network mapping qualitative analysis of local decision-makers from nine different sectors in North Carolina documents the complex, cross-sector local decision landscape in response to the COVID-19 pandemic. Most notably, this analysis highlights the expanded roles of decision-makers across sectors in the pandemic response, the key types of decisions faced, and how decision-makers relied on collaboration and the guidance of LHDs to respond to these new challenges. Understanding this decision landscape serves to inform public health researchers and practitioners about who is involved in decision-making related to community health and how. Knowing this can support communities in collaborating to improve organizational decision-making processes with community and population health in mind. It also underscores the need for public health infrastructure to improve information dissemination, priority setting, and alignment in response to future crises and other complex health challenges.

## Data availability statement

The datasets generated for this study are not publicly available due to data confidentiality. Investigators interested in accessing this dataset for future research may do so under the following conditions: (1) IRB approval has been obtained from the institution covering the investigator, (2) data security procedures ensuring patient privacy have been demonstrated by the investigator, and (3) a data use agreement is completed by UNC and the outside investigator. Final datasets for analysis will not include any identifying information.

## Ethics statement

The studies involving human participants were reviewed and approved by UNC Institutional Review Board. Written informed consent for participation was not required for this study in accordance with the national legislation and the institutional requirements.

## Author contributions

Data curation: CB and KJ. Formal analysis: CB, KJ, and HH. Funding acquisition: MP, MM, JS, and KH. Investigation: KJ and KH. Methodology: CB, KJ, MP, and KH. Project admin: KJ. Supervision: MP and KH. Validation, visualization, and writing–original draft: CB. All authors contributed to the conceptualization of this study, writing–review and editing, and approved the submitted version.

## Funding

This research was supported by the National Center for Advancing Translational Sciences (NCATS), National Institutes of Health, Grant No. UL1TR002489, and the Council of State and Territorial Epidemiologists and Centers for Disease Control Cooperative Agreement No. NU38OT000297. CBB is additionally supported by a NIH Cancer Care Quality Training Program grant, UNC-CH, Grant No. T32-CA-116339. Funders did not have any role in the study design; collection, management, analysis, and interpretation of the data; writing of the manuscript; or the decision to submit the report for publication.

## Conflict of interest

JS reported receiving compensation from Georgia Institute of Technology and Zoetis, Inc. in the prior 12 months. The remaining authors declared that the research was conducted in the absence of any commercial or financial relationships that could be construed as a potential conflict of interest.

## Publisher's note

All claims expressed in this article are solely those of the authors and do not necessarily represent those of their affiliated organizations, or those of the publisher, the editors and the reviewers. Any product that may be evaluated in this article, or claim that may be made by its manufacturer, is not guaranteed or endorsed by the publisher.

## References

[1] World Health Organization. Director-General's opening remarks at the media briefing on COVID-19-11 March 2020. Geneva: World Health Organization (2020).

[2] Cucinotta D Vanelli M WHO. Declares COVID-19 a pandemic. Acta Biomed. (2020) 91:157–60. 10.23750/abm.v91i1.9397 32191675 PMC7569573

[3] WHO. WHO Coronavirus (COVID-19) Dashboard. Availableonline at: https://covid19.who.int/. Published 2022. Updated 1/6/2022 (accessed January 6, 2022).

[4] Sturmberg JP Martin CM. COVID-19 – how a pandemic reveals that everything is connected to everything else. J Eval Clin Pract. (2020) 26:1361–7. 10.1111/jep.13419 32633056 PMC7362160

[5] Wernli D Tediosi F Blanchet K Lee K Morel C Pittet D. A complexity lens on the COVID-19 pandemic. Int J Health Policy Manag. (2021). 10.34172/ijhpm.2021.55. [Epub ahead of print]. 34124870 PMC9818100

[6] Baugh Littlejohns L Baum F Lawless A Freeman T. The value of a causal loop diagram in exploring the complex interplay of factors that influence health promotion in a multisectoral health system in Australia. Health Res Policy Syst. (2018) 16:126. 10.1186/s12961-018-0394-x 30594203 PMC6310960

[7] Cilenti D Issel M Wells R Link S Lich KH. System dynamics approaches and collective action for community health: an integrative review. Am J Community Psychol. (2019) 63:527–45. 10.1002/ajcp.12305 30706946

[8] Hassmiller Lich K Kuhlberg J. Engaging Stakeholders in Mapping and Modeling Complex System Structure to Inform Population Health Research and Action (Chapter 9). New York, NY: Oxford University Press. (2020). p. 119–33.

[9] Koleros A Mulkerne S Oldenbeuving M Stein D. The actor-based change framework: a pragmatic approach to developing program theory for interventions in complex systems. Am J Eval. (2018) 41:34–53. 10.1177/1098214018786462

[10] Urry J. The complexity turn. Theory Cult Soc. (2005) 22:1–14. 10.1177/0263276405057188

[11] Siegenfeld AF Bar-Yam Y. An introduction to complex systems science and its applications. Complexity. (2020) 2020:6105872. 10.1155/2020/6105872

[12] Emshoff JG Darnell AJ Darnell DA Erickson SW Schneider S Hudgins R. Systems change as an outcome and a process in the work of community collaboratives for health. Am J Community Psychol. (2007) 39:255–67. 10.1007/s10464-007-9110-7 17410424

[13] Czeisler M Tynan MA Howard ME Honeycutt S Fulmer EB Kidder DP. Public attitudes, behaviors, and beliefs related to COVID-19, stay-at-home orders, nonessential business closures, and public health guidance - United States, New York City, and Los Angeles, May 5-12, 2020. MMWR Morb Mortal Wkly Rep. (2020) 69:751–8. 10.15585/mmwr.mm6924e1 32555138 PMC7302477

[14] Vardavas C Odani S Nikitara K Banhawi HE Kyriakos C Taylor L. Public perspective on the governmental response, communication and trust in the governmental decisions in mitigating COVID-19 early in the pandemic across the G7 countries. Prev Med Rep. (2021) 21:101252. 10.1016/j.pmedr.2020.101252 33364149 PMC7753973

[15] Vielot NA Horney JA. Can merging the roles of public health preparedness and emergency management increase the efficiency and effectiveness of emergency planning and response? Int J Environ Res Public Health. (2014) 11:2911–21. 10.3390/ijerph110302911 24619123 PMC3987012

[16] Davis MV MacDonald PD Cline JS Baker EL. Evaluation of public health response to hurricanes finds North Carolina better prepared for public health emergencies. Public Health Rep. (2007) 122:17–26. 10.1177/003335490712200103 17236604 PMC1802115

[17] Kegler MC Swan DW. An initial attempt at operationalizing and testing the community coalition action theory. Health Educ Behav. (2011) 38:261–70. 10.1177/1090198110372875 21393621

[18] NCDHHS. North Carolina COVID-19 Dashboard. Kumu (2022).

[19] Hsieh HF Shannon SE. Three approaches to qualitative content analysis. Qual Health Res. (2005) 15:1277–88. 10.1177/1049732305276687 16204405

[20] *MAXQDA* [computer program]. Berlin, Germany: VERBI Software. (2019).

[21] Henneman EA Lee JL Cohen JI. Collaboration: a concept analysis. J Adv Nurs. (1995) 21:103–9. 10.1046/j.1365-2648.1995.21010103.x 7897060

[22] Gray B. Collaborating: Finding Common Ground for Multiparty Problems. 1st ed ed San Francisco: Jossey-Bass. (1989).

[23] Tong A Sainsbury P Craig J. Consolidated criteria for reporting qualitative research (COREQ): a 32-item checklist for interviews and focus groups. Int J Qual Health Care. (2007) 19:349–57. 10.1093/intqhc/mzm042 17872937

[24] *Kumu Inc*. [computer program]. North Carolina Department of Health and Human Services (NCDHHS) (2021).

[25] Wijenberg E Wagemakers A Herens M Hartog FD Koelen M. The value of the participatory network mapping tool to facilitate and evaluate coordinated action in health promotion networks: two Dutch case studies. Glob Health Promot. (2019) 26:32–40. 10.1177/1757975917716923 28832266 PMC6755660

[26] Pinfold V Sweet D Porter I Quinn C Byng R Griffiths C. Improving Community Health Networks for People With Severe Mental Illness: A Case Study Investigation. Southampton (UK): NIHR Journals Library. (2015). Available online at: https://www.ncbi.nlm.nih.gov/books/NBK276549/25741571

[27] Ostrom E. Beyond markets and states: polycentric governance of complex economic systems. Am Econ Rev. (2010) 100:641–72. 10.1257/aer.100.3.641

[28] Gupta R. Enhancing community partnerships during a public health emergency: the school-located vaccination clinics model in Kanawha County, WV during the 2009 influenza A (H1N1) pandemic. W V Med J. (2011) 107:28–34. 22235709

[29] Simo G Bies AL. The role of nonprofits in disaster response: an expanded model of cross-sector collaboration. Pub Admin Rev. (2007) 67:125–42. 10.1111/j.1540-6210.2007.00821.x

[30] Uppal A Silvestri DM Siegler M Natsui S Boudourakis L Salway RJ. Critical care and emergency department response at the epicenter of the COVID-19 pandemic. Health Aff (Project Hope). (2020) 39:1443–9. 10.1377/hlthaff.2020.00901 32525713

[31] Hoover AG Heiger-Bernays W Ojha S Pennell KG. Balancing incomplete COVID-19 evidence and local priorities: risk communication and stakeholder engagement strategies for school re-opening. Rev Environ Health. (2021) 36:27–37. 10.1515/reveh-2020-0092 33001857 PMC7933073

[32] Ryan B Coppola D Canyon D. Incremental community-based exit strategies for initiating and removing COVID-19 lockdowns. In: Daniel K. Inouye Asia-Pacific Center for Security Studies. Honolulu, HI: Daniel K. Inouye Asia-Pacific Center for Security Studies (2020).

[33] Hyder A Trinh A Padmanabhan P Marschhausen J. Wu A Evans A. COVID-19 surveillance for local decision making: an academic, school district, and public health collaboration. Public Health Rep. (2021) 136:403–12. 10.1177/00333549211018203 33979558 PMC8203033

[34] Storeng KT de Bengy Puyvallée A. The smartphone pandemic: how big tech and public health authorities partner in the digital response to Covid-19. Glob Public Health. (2021) 16:1482–98. 10.1080/17441692.2021.1882530 33602063

[35] Lane ECA Tran AA Graulty CJ Bumsted T. Rapid mobilization of medical students to provide health care workers with emergency childcare during the COVID-19 pandemic. Acad Med. (2021) 96:1302–5. 10.1097/ACM.0000000000004115 33788791 PMC8378428

[36] Panneer S Kantamaneni K Pushparaj RRB Shekhar S Bhat L Rice L. Multistakeholder participation in disaster management-the case of the COVID-19 pandemic. Healthcare (Basel). (2021) 9:203. 10.3390/healthcare9020203 33668669 PMC7918841

[37] Loewenson R Colvin CJ Szabzon F Das S Khanna R Coelho VSP. Beyond command and control: A rapid review of meaningful community-engaged responses to COVID-19. Glob Pub Health. (2021) 16:1–15. 10.1080/17441692.2021.1900316 33734007

[38] Bruno B Hurwitz HM Mercer M Mabel H Sankary L Morley G. Incorporating stakeholder perspectives on scarce resource allocation: lessons learned from policymaking in a time of crisis. Camb Q Healthc Ethics. (2021) 30:390–402. 10.1017/S0963180120000924 33764294

[39] DeSalvo KB O'Carroll PW Koo D Auerbach JM. Monroe JA. Public health 30: time for an upgrade. Am J Pub Health. (2016) 106:621–2. 10.2105/AJPH.2016.303063 26959263 PMC4816012

[40] Torfing J Peters GB Pierre J Sørensen E. Interactive Governance: Advancing the Paradigm, Chapter 7 - Metagovernance: The art of governing interactive governance. Oxford University Press. (2012). p. 122–44.

[41] Apostolopoulos Y. Bridging the Divide: Where Complex Systems Science Meets Population Health Science (Chapter 1). New York, NY: Oxford University Press (2020).

[42] Mattessich PW Rausch EJ. Cross-sector collaboration to improve community health: a view of the current landscape. Health Aff (Project Hope). (2014) 33:1968–74. 10.1377/hlthaff.2014.0645 25367992

[43] Towe VL Leviton L Chandra A Sloan JC Tait M Orleans T. Cross-sector collaborations and partnerships: essential ingredients to help shape health and well-being. Health Aff. (2016) 35:1964–9. 10.1377/hlthaff.2016.0604 27834234

[44] DeSalvo KB Wang YC Harris A Auerbach J Koo D. O'Carroll P. Public health 30: a call to action for public health to meet the challenges of the 21st century. Prev Chronic Dis. (2017) 14:E78. 10.5888/pcd14.170017 28880837 PMC5590510

[45] 10 Essential Public Health Services EPHS Toolkit. Public Health National Center for Innovations. (2020).

[46] Jarrah S Khaldun J Sellers K Rich N. Bringing the essential public health services to life. J Public Health Manag Pract. (2021) 27:97–8. 10.1097/PHH.0000000000001298 33239531

[47] Romano SD Blackstock AJ Taylor EV Felix SEB Adjei S Singleton CM. Trends in racial and ethnic disparities in COVID-19 hospitalizations, by region — United States, March–December 2020. Morb Mort Wkly Rep. (2021) 70:560–5. 10.15585/mmwr.mm7015e2 33857068 PMC8344991

[48] Dickinson KL Roberts JD Banacos N Neuberger L Koebele E Hartigan E. Structural racism and the COVID-19 experience in the United States. Health Secur. (2021) 19:S14–26. 10.1089/hs.2021.0031 34076499

[49] Galaitsi SE Cegan JC Volk K Joyner M Trump BD Linkov I. The challenges of data usage for the United States' COVID-19 response. Int J Inf Manage. (2021) 59:102352. 10.1016/j.ijinfomgt.2021.102352 33824545 PMC8017563

[50] Hester JA Stange PV Seeff LC Davis JB Craft CA. Towards Sustainable Improvements in Population Health: Overview of Community Integration Structures and Emerging Innovations in Financing. US: Centers for Disease Control and Prevention. (2015). p. 1–16.

[51] Stone KW Kintziger KW Jagger MA Horney JA. Public health workforce burnout in the COVID-19 response in the U.S. Int J Environ Res Public Health. (2021) 18:4369. 10.3390/ijerph18084369 33924084 PMC8074254

[52] Bourgeault IL Maier CB Dieleman M Ball JE Mackenzie A Nancarrow S. The COVID-19 pandemic presents an opportunity to develop more sustainable health workforces. Hum Resour Health. (2020) 18:83. 10.1186/s12960-020-00529-0 33129313 PMC7602762

